# Unusual Morphological Changes of a Novel Wrinkled Bacterium Isolated from the Rice Rhizosphere Under Nutrient Stress

**DOI:** 10.3390/life15091337

**Published:** 2025-08-23

**Authors:** Young Ryun Chung, Jung Eun Lee, Zubair Aslam, Eu Jin Chung, Kwang Hee Lee, Byung Ho Kang, Ajmal Khan, Sarbjeet Niraula, Woo-Suk Chang

**Affiliations:** 1 Plant Molecular Biology and Biotechnology Research Centre, Gyeongsang National University, Jinju 52828, Republic of Korea; 2 Department of Research & Technology Development, JGreen Inc., Jinju 52840, Republic of Korea; genetist@hanmail.net; 3 Department of Agronomy, University of Agriculture, Faisalabad 38040, Pakistan; zauaf@hotmail.com; 4 Nakdonggang National Institute of Biological Resources, Sangju 37242, Republic of Korea; eujenee@nnibr.re.kr; 5 Department of Plant Science and Landscape Architecture, University of Connecticut, Storrs, CT 06269, USA; kwang_hee.lee@uconn.edu; 6 State Key Laboratory of Agrobiotechnology, School of Life Sciences, Centre for Cell and Developmental Biology, The Chinese University of Hong Kong, Hong Kong, China; bkang@cuhk.edu.hk; 7 Department of Biotechnology, Bacha Khan University, Charsadda 24420, Pakistan; ajmalkhanbbt@gmail.com; 8 Department of Surgery, Feinberg School of Medicine, Northwestern University, Chicago, IL 60611, USA; sarbjeet.niraula@northwestern.edu; 9 Department of Biology, University of Texas at Arlington, Arlington, TX 76019, USA

**Keywords:** rice rhizosphere, electron microscopy, 16S rRNA sequencing, whole-genome sequencing, *Rugositalea oryzae*, environmental stress

## Abstract

Bacterial cell morphology might result from natural selection to gain a competitive advantage under environmentally stressful conditions such as nutrient limitation. In nutrient-limited conditions, a higher surface-to-volume ratio is crucial for cell survival because it allows for a more efficient exchange of nutrients and waste products. A bacterial strain YC6860^T^ isolated from the rhizosphere of rice (*Oryza sativa* L.) showed pleomorphic behavior with smooth cell morphology and wrinkled surface rods depending upon nutritional conditions. Based on scanning and transmission electron microscopy studies, we hypothesized that the surface-to-volume ratio of cells would increase with decreasing nutrient concentrations and tested this quantitatively. The transition from smooth to wrinkled cell surface morphology could be one of the adaptation strategies by which YC6860^T^ maximizes its ability to access available nutrients. To characterize the properties of the wrinkled strain, we performed taxonomic and phylogenetic analyses. 16S rRNA gene sequencing results showed that the strain represented a novel, deep-rooting lineage within the order *Rhizobiales* with the highest similarity of 94.2% to *Pseudorhodoplanes sinuspersici* RIPI 110^T^. Whole-genome sequencing was also performed to characterize its genetic features. The low phylogenetic and genetic similarity is probably related to the wrinkled morphology of the strain. Therefore, we propose that the strain YC6860^T^ might belong to a new genus and species, named *Rugositalea oryzae*. In addition, taxonomic analysis showed that YC6860^T^ is Gram-negative, aerobic, and rod-shaped with regular surface wrinkles under nutrient-limiting conditions, resembling a delicate twist of fusilli, with groove depths of 48.8 ± 3.7 nm and spacing of 122.5 ± 16.9 nm. This unique cell structure with regular rugosity could be the first finding that has not been reported in the existing bacterial morphology.

## 1. Introduction

Bacteria have evolved over a long period of time to adapt to stressful environments by changing morphology, which encompasses shape, arrangement, and size. Different shapes allow bacteria to optimize nutrient acquisition, motility, and resistance to various stressors. Understanding bacterial morphology is essential for identifying and classifying species, as well as for comprehending their ecological roles. Various bacterial cell morphologies, such as rods, cocci, filaments, star-like shapes, helical/spiral forms, and prosthecate structures [1,2,3,4,5], likely arise from the evolution of bacteria and thus represent the basis of bacterial classification [6,7]. Morphological alterations of bacterial cells under environmental fluctuations in nutrient availability, temperature, space availability, and moisture content have been extensively studied [8,9,10,11,12]. Nutrient limitation occurs most often in an environment with active microbial competition. Under such adverse conditions, bacteria have evolved competitive mechanisms by which they produce digestive enzymes or other protective and storable metabolites to outcompete their neighbors [13]. The slow-down of cell division, switching to fermentation, and alteration in cell morphology are also important metabolic and phenotypic strategies of survival [6,13]. The competition could induce morphological changes in bacteria, such as curved, elongated stalks and branched and filamentous shapes, to increase attachment to the host surface or enhance nutrient uptake. The role of stalk lengthening in *Caulobacter crescentus* is to enhance nutrient absorption in oligotrophic aquatic environments with relatively low nutrient concentrations [8,14,15]. In addition, the altered cell size and shape to effectively improve nutrient uptake under nutrient deficiencies has been documented in model species such as *Escherichia coli* and *Bacillus subtilis* [2,7,16].

Nutrient availability is the strongest factor affecting bacterial growth and survival in the rhizosphere. The rhizosphere is an energy-rich zone containing up to 40% of host photosynthetic carbon, and therefore, microbes compete for space on the root surface and nutrients within the rhizosphere. This competition can be intense, leading to the evolution of various microbial strategies for survival and dominance. Key nutrients associated with organic matter in the rice rhizosphere include organic carbon, nitrogen, phosphorus, potassium, sulfur, and essential micronutrients such as iron, zinc, and manganese. However, the availability and concentration of these nutrients vary considerably depending on geographical location, soil texture, pH, and cultivation practices. For instance, in the Indo-Gangetic Plains of India, the total organic carbon (TOC) in the surface 0–15 cm soil layer has been reported to be approximately 6.8 g/kg [17]. In contrast, a study conducted in Chinese paddy soils indicated a higher organic carbon content, highlighting the influence of local environmental factors [18]. Similarly, in a subtropical rice-growing region of China, the rhizosphere soil was characterized by 7.5% clay, 68.4% silt, and 24.1% sand, with 15.6 g/kg organic carbon, 1.6 g/kg total nitrogen, and 0.5 g/kg total phosphorus, while the pH was 5.8 (2:5 soil-to-water ratio) [19]. These findings underscore the spatial heterogeneity and physicochemical complexity of rice rhizosphere soils across different agroecological zones.

The microbial interactions in the rhizosphere are associated with various types of plant exudates consisting of low- and high-molecular-weight compounds [20]. Certain flavonoids are known to initiate the symbiotic process between legumes and nitrogen-fixing bacteria, while others attract beneficial microbes (e.g., plant growth-promoting rhizobacteria (PGPR)) that could suppress harmful microbes or induce host resistance against plant pathogens or abiotic stresses [21,22]. In particular, microbial competition for nutrients within the rhizosphere could be triggered by the elevated level of microbial population, plant genotypes, and types of root exudates [23,24,25]. In nutrient deficiency, the surface area-to-volume (S/V) ratio plays a critical role, as nutrient uptake may rely on the extent of the exposed surface area. A high surface-area-to-volume (S/V) ratio is crucial for cell survival, under nutrient-limited conditions, because it allows for efficient exchange of resources and waste products. Smaller or wrinkled cells have a larger surface area relative to their volume, enabling them to take in nutrients and expel waste more quickly through diffusion.

In order to achieve the large S/V ratio, bacterial cells may limit their size or transform their regular morphology to filamentous or prosthecate shapes [7]. For instance, the S/V ratio of rod-shaped bacterial cells remains constant under growth, although it decreases in spherical-shaped bacteria [26,27]. Peptidoglycans and cytoskeleton consisting of actin filaments and fatty acids are key factors in the morphological transition of bacterial cells [1,10,28]. The proteins involved in bacterial cell division, particularly a polymerized tubulin-like protein FtsZ, and shape-determining proteins, also play a critical role in shaping the cell. Variations in these proteins or their regulation can lead to diverse bacterial morphologies, and the bacteria can even adapt their shape in response to environmental changes [29,30]. In nutrient-rich media, the nutrients act upon metabolic sensors, which increase the cell size via FtsZ assembly inhibition, or increase the Z-period length before cytokinesis. In nutrient-poor media, the protein expression was decreased, randomly dispersed and had little effect on FtsZ assembly, resulting in reduction in the average cell size [1,16,31]. Recently, the mechanisms underlying the evolution of altered bacterial shape have been elucidated via the analysis of genome sequence data [2,32]. By mapping morphological phenotypes onto phylogenetic trees, we were able to gain an insight into how bacterial shapes evolved. Some morphologies such as helical and filamentous types, are spread all over the bacterial domain, indicating that they have evolutionally several origins [2]. However, the same morphology is clustered together in a specific region of the phylogenetic tree, suggesting that this morphology came from a shared ancestor [2].

Morphological plasticity in bacteria is known to occur under environmental stress. However, its mechanistic basis and ecological relevance, particularly in newly identified rhizosphere strains such as YC6860^T^, remain poorly understood. This study addresses this gap by linking nutrient-dependent morphological changes to shifts in the S/V ratio, genomic features, and potential adaptive strategies in the rice rhizosphere. Therefore, we hypothesized that the altered cell morphology from smooth to wrinkled rods of bacterial strain YC6860^T^ increased the S/V ratio of cells under nutritional stress. To test this hypothesis, we set the following objectives: (i) to characterize the morphological changes under varying nutrient conditions, (ii) to determine the physiological significance of these changes, and (iii) to establish the taxonomic and genomic identity of the strain. The hypothesis was proved by phenotypic observations of the strain cultivated under different nutrient concentrations using electron microscopy. Not only was the novelty of this strain characterized by a polyphasic approach, but also its full genome sequence was analyzed. Genomic analysis revealed unique gene clusters potentially involved in cell envelope remodeling and stress adaptation. Comparative genomics further supported the distinct taxonomic position of YC6860^T^ within its clade. These findings suggest a strong link between morphological plasticity and ecological fitness under nutrient-limited conditions.

## 2. Materials and Methods

### 2.1. Isolation of Bacterial Strain

Soil samples for bacterial isolation were collected from the rhizosphere of rice paddy fields managed under no-tillage practices for 5 years, located at Gyeongsang National University farm (Daegok valley, 35°14′21″ N, 128°13′23″ E), Jinju, Korea. To isolate bacteria from the samples, 1 g of soil was added to 10 mL of buffer solution (50 mM phosphate buffer, pH 7.0) and half of the soil suspension was sonicated for 15 s with an electronic homogenizer (Bandelin Sonoplus, Berlin, Germany) at 260 W/cm^2^. Serial dilutions were obtained after mixing both sonicated and non-sonicated portions, and 100 µL diluted (10^−3^–10^−5^) aliquots were spread on half-strength (0.5) R2A agar plates in large Petri dishes (150 mm in diameter). These agar media supplemented with amphotericin B (50 µg/mL) to inhibit fungal growth, and 40% (*w*/*v*) soil extract, a nutrient-poor medium, were incubated at 28 °C for six weeks to selectively cultivate slow-growing, oligotrophic bacteria. The extended incubation period and low-nutrient environments promote the growth of bacteria adapted to such conditions, while inhibiting the proliferation of faster-growing, more nutrient-demanding bacteria. The small (less than 1 mm) bacterial colonies were selected on the basis of morphology and the isolates were purified and sub-cultured on 0.5 R2A. Strain YC6860^T^ was isolated and purified from the rhizosphere of the no-tillage (Z2) rice field and stored at −80 °C in 20% (*w*/*v*) glycerol stocks for further tests [33].

### 2.2. Bacterial Culture Preparation

Strain YC6860^T^ was cultured in various culture media with different nutrient compositions such as tryptic soy broth (TSB), tryptic soy agar (TSA), R2A, Luria Bertani (LB), and nutrient agar (NA) to determine the optimal medium for growth. All these media were purchased from Difco and diluted from one-tenth (0.1) to half-strength (0.5) using sterile distilled water. During the first set of experiments, the growth of the strain was better in TSA or TSB media compared with other media, and therefore, these were selected for further experiments. Growth was measured by inoculating two to three loops of colonies developed on 0.5 R2A agar plates for one month at 28 °C into 100 mL 0.1~0.5 TSB media in 250 mL Erlenmeyer flasks, and incubated at 28 °C with shaking (140 rpm) for the indicated periods. The optical density of a bacterial culture at 600 nm (OD600) was measured using a spectrophotometer (X-ma 1200, Human Corporation, Seoul, Republic of Korea). The cells cultivated in 0.1~0.5 TSB media were collected by centrifugation and prepared for microscopic observation. Bacterial cells grown for 15 days on 0.1 TSA were characterized biochemically and physiologically. All experiments were conducted with at least three biological replicates, and control cells (e.g., non-transferred cells) were maintained in parallel during transfer experiments.

### 2.3. Bacterial Cell Morphology

Cell morphology of strain YC6860^T^ was observed under a Nikon light microscope (Tokyo, Japan) at 1000× after Gram-staining. The motility was determined using cells grown on 0.5 R2A agar plates for seven days at 28 °C by phase-contrast microscopy. Flagella were observed via transmission electron microscopy (TEM) (Jeol, JEM 2010, Tokyo, Japan) after negative staining of specimens with 2% phosphotungstic acid on 200-mesh formvar-coated copper grids. The ultrathin sections of cells prepared using an ultramicrotome (Leica EM UC6, Nussloch, Germany) were double-stained with 2.0% uranyl acetate and lead citrate solution and used for ultrastructural observations. The voxel resolution was 2 × 2 × 12 nm along the x-, y-, and z-axes, respectively. Scanning electron microscopy (SEM) was performed using bacterial cells cultivated in different culture broths at different concentrations (0.1~0.5 TSB), then recovered by centrifugation (8000× *g* at 4 °C) for 10 min and washed twice with 0.1 M phosphate-buffered saline (PBS, Oxide). The cell pellets were suspended in 2.5% glutaraldehyde and stored at 4 °C for 4 h. Samples were rinsed three times with PBS, post-fixed in 1% osmium tetroxide for 1 h, washed three times with PBS, dehydrated in ethanol solutions (60, 70, 80, and 90%, twice at 100%) for 5 min, with 5 min intervals between treatments. The dehydrated samples were treated with 1 mL hexamethyldisilizine, dried overnight, and coated with gold-palladium in an SEM coating unit E5000 (Polaron Equipment Ltd, Hertfordshire, England) for observation under a scanning electron microscope (JEOL JSM-6380LV, Tokyo, Japan) at the High-Tech Materials Analysis Core Facility, GNU [34].

### 2.4. Monitoring of Bacterial Cell Shape Under Nutrient Stress

To determine the primary effect of nutrient concentration on cell morphology and growth, the strain YC6860^T^ was first cultured in 0.5 TSB for 15 days as described above and the same cell pellets were harvested by centrifugation (8000× *g* at 4 °C) for 10 min and washed twice with 0.1 TSB. The washed cells were transferred to 0.1 TSB and cultivated until 22 days after the initial inoculation of the strain. Samples of bacterial cells were collected at 7 and 16 days after cell transfer for observation of cell morphology and growth, and the measurement of total organic carbon (TOC) concentration. Harvest and transfer were performed aseptically to prevent contamination of bacterial cells, while the consistent conditions, including temperature (28 °C), aeration (140 rpm), and neutral pH (~7.0), were maintained during the transfer.

### 2.5. Measurement of the Surface Area-to-Volume (S/V) Ratio of Bacterial Cells

To measure the S/V ratio of bacterial cells cultured in TSB containing various nutrient concentrations (0.1~0.5 TSB) for 15 days at 28 °C, serial sectioning and reconstruction of 3D models using TEM were carried out as described previously [35]. Briefly, sample blocks for TEM were sliced into serial section ribbons (120 nm thickness) and stained with uranyl acetate solution (3 min) and lead citrate solution (6 min). Images from serial sections were stacked into MRC format files and bacterial cells were outlined to prepare 3D models using the IMOD software package (version 4.9). For TEM analyses, approximately 50 low-magnification images (250–3000×) and more than 100 high-magnification images (15,000–30,000×) were captured and examined to choose regions for 3D reconstruction. Morphometric information of bacterial cells was acquired with the imodinfo command in the package and the S/V ratios were calculated using the Microsoft Excel program. In each sample, 8–30 bacterial cells were randomly selected to calculate their volume and surface. For round cells grown in 0.5 TSB media, analyzing 8–15 cells was sufficient to calculate S/V ratios due to their uniform morphology. In contrast, for elongated cells cultured in lower-concentration TSB media, 20–30 cells with varying orientations were examined to minimize errors associated with their anisotropic shape. This experiment was repeated to confirm the changes in the S/V ratio and all images are representative of at least triplicate biological samples. The relationship between TSB concentrations and S/V ratios of bacterial cells was subjected to regression analysis.

### 2.6. Determination of Total Organic Carbon

The TOC concentration in the culture supernatant of strain YC6860^T^ was determined via the 680 °C combustion–infrared method using a Shimadzu carbon analyzer (TOC-VCPN, Kyoto, Japan). The carbon dioxide generated by oxidation was detected using an infrared gas analyzer according to the manufacturer’s manual. The culture supernatant was prepared by centrifugation (5000× *g*, 15 min) of bacterial cells cultured for 22 days at 28 °C. Triplicates of each sample were analyzed.

### 2.7. Phylogenetic Analysis of 16S rRNA Gene Sequences

The genomic DNA was extracted from the strain YC6860^T^ using a commercial DNA extraction kit (Core Biosystem, Seoul, Republic of Korea). The 16S rRNA gene was PCR-amplified from purified genomic DNA using a set of primers 27F and 1492R [36], and the purified PCR product (1466 bps) was sequenced by GenoTech Inc. (Daejeon, Republic of Korea). The 16S rRNA gene sequences were compiled using SeqMan software (Lasergene v16, DNASTAR) and the sequences of related taxa were obtained from the GenBank database. The chimera in the sequence of strain was checked using the CHECK_CHIMERA program in the Ribosomal Database Project (RDP) [37]. The phylogenetic position of strain YC6860^T^ was determined by comparing its 16S rRNA gene sequence with sequences of related taxa collected from NCBI and EzBioCloud server (http://www.ezbiocloud.net) [38]. Multiple alignments were performed using the CLUSTAL_X program [39] and a BioEdit program was used for gap editing [40]. The evolutionary distances were calculated using the Kimura two-parameter model [41]. The phylogenetic tree was constructed using a neighbor-joining method [42] and maximum-parsimony [43] in a MEGA 4 program (version 4) [44] with bootstrap values based on 1000 replications [45].

### 2.8. Physiological and Biochemical Tests

API ZYM, API 20E, and API 20NE kits were used for physiological and biochemical characterization of strain YC6860^T^. API ZYM strips were read after a 5 h incubation. The assimilation of a single substrate was determined using the API 20NE kit at 30 °C after 24 h of incubation. The bacterial cells were grown at different temperatures ranging from 4 °C to 40 °C. Anaerobic growth was tested at 28 °C by pouring a thick layer of vaspar (50% petrolatum, 50% paraffin) on the surface of inoculated semi-solid half-strength R2A agar (1.0% agar) in 35 mL screw-capped glass tubes [46]. Growth in sodium chloride was observed at 0–5% (*w*/*v*), while growth at pH 5–10 was determined after 7 days of incubation. Oxidase and catalase activities were determined according to the method of Cappuccino and Sherman [47]. Gram-staining was carried out using a Gram-staining kit (BD) according to the manufacturer’s instructions. All experiments were conducted with at least three biological replicates.

### 2.9. Chemotaxonomic Characterization

The G + C content of the chromosomal DNA was determined after purification and extraction of genomic DNA as previously reported [48]. It was then degraded enzymatically into nucleosides and the G + C contents were determined via reverse-phase HPLC [49]. The quinone system was determined by TLC [50]. The fatty acid profile was determined by incubating the strain YC6860^T^ either in R2A or 0.1 TSB and 0.5 TSB at 28 °C for 10 days. Cellular fatty acids were saponified, methylated, and extracted according to the standard protocol of Sasser [51], and their composition was determined using GC (6890; Hewlett Packard, Palo Alto, CA, USA) and a microbial identification software package (Microbial ID, Newark, DE, USA).

### 2.10. Whole-Genome Sequences of Strain YC6860^T^

Complete genome of strain YC6860^T^ was sequenced by extracting its high-quality genomic DNA with the blood and cell culture DNA minikit (Qiagen, Germantown, MD, USA). The genome of YC6860^T^ was sequenced and characterized using Illumina GAIIx sequencer (San Diego, CA, USA) with 100 bp paired-end libraries from Chun-Lab, Inc. (Seoul, Republic of Korea). Using this platform, we typically achieved a read coverage of approximately 50X. GS Assembler 2.3 (Roche Diagnostics, Branford, CT, USA) and Codon Code Aligner 3.0 were used to assemble all sequence data and the gaps between all the contigs were filled by PCR amplification. The coding sequences (CDSs) were determined with Glimmer 3.02. Transfer RNAs (tRNAs) were determined by tRNAscan-SE [52], while the ribosomal RNA (rRNA) was located using HMMER with EzBiocloud-e-rRNA profiles [38,53]. The CDSs were compared by rpsBLAST (BLAST+ v2.6.0) and NCBI reference sequences (Ref-Seq) to catalytic families (Catfam) and the NCBI COG. The functional annotation of the CDSs was determined by employing the SEED database [54,55].

### 2.11. Accession Numbers of 16S rRNA Gene and Genome Sequences of YC6860^T^

The nucleotide sequence of the 16S rRNA gene and the complete genome sequence of strain YC6860^T^ were submitted to NCBI and assigned the accession numbers GQ369128 (16S rRNA gene) and CP007440 (whole genome), deposited under BioProject PRJNA238489 and BioSample SAMN04521129.

## 3. Results

### 3.1. Growth and Morphology of the Strain YC6860^T^

**Figure 1:**
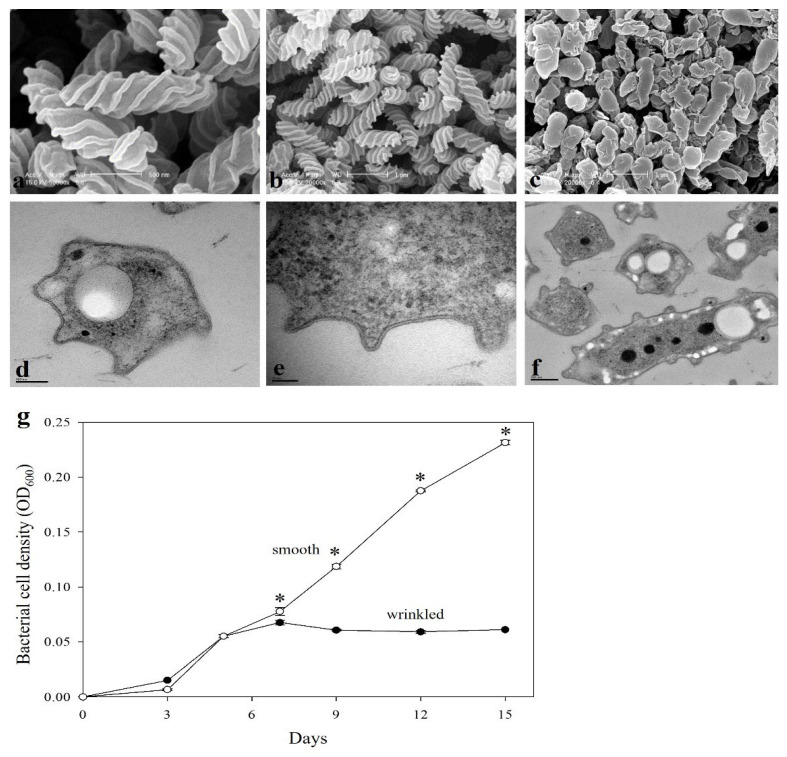
The effects of nutrient media on the growth of *Rugositalea oryzae* YC6860^T^. Bacterial cells grown in 0.1 TSB (**a**,**b**) and 0.5 TSB (**c**) on a rotary shaker at 28 °C for 15 days were observed under an SEM. Wrinkled cells were observed under a TEM (**d**–**f**). White bars represent scales of 0.5 µm, 1 µm, and 1 µm in SEM images (**a**–**c**), respectively, while black bars in TEM images (**d**–**f**) indicate scales of 100 nm, 50 nm, and 200 nm, respectively. Bacterial cell density in 0.1 TSB (closed circles) and 0.5 TSB (open circles) was observed at different time intervals (**g**). The open circles show bacterial growth in smooth cells, while the closed circles indicate bacterial growth in wrinkled cells with time (days). Asterisks indicate statistically significant differences (*p* ≤ 0.01) by a *t*-test.

During the analysis of bacterial community in the rhizosphere of rice cultivated under conventional and no-tillage practices, the strain YC6860^T^ was isolated from the rice rhizosphere in the reproductive phase at a no-tillage paddy field [33]. Under SEM, the rod cells showed unique wrinkled features comprising grooves and margins uniformly distributed over the bacterial surface (Figure 1a,b). However, the cells grown in 0.5 TSB for 7 days showed irregular rod or cocci shape without regular wrinkles, which we consider as smooth surfaces (Figure 1c). Under TEM, the cell structures, including the cell wall and membrane, were altered in the grooves and margins of the wrinkles (Figure 1d–f). To confirm this unique change in cell shape, the strain YC6860^T^ was cultured in nutrient media at two different concentrations and the morphology was compared. Under high-nutrient conditions (0.5 TSB), the log phase begins on day 3, and bacterial growth continues until day 15. In contrast, under low-nutrient conditions (0.1 TSB), growth ceases by day 5, followed by entry into the stationary phase (Figure 1g). The strain grown in high-nutrient media (0.5 TSB) showed smooth cells shapes, while the cells grown in low-nutrient media (0.1 TSB) were wrinkled (Figure 1a–f). The morphology of this strain cultivated in 0.1 LB and 0.5 LB media was also similar in pattern to the strain under 0.1 TSB and 0.5 TSB (Supplementary Figure S1a,b). This result suggested that nutrient concentration of the culture media could be the main factor underlying the change in shape from smooth to wrinkled form.

### 3.2. Transition from Smooth to Wrinkled Shape

**Figure 2:**
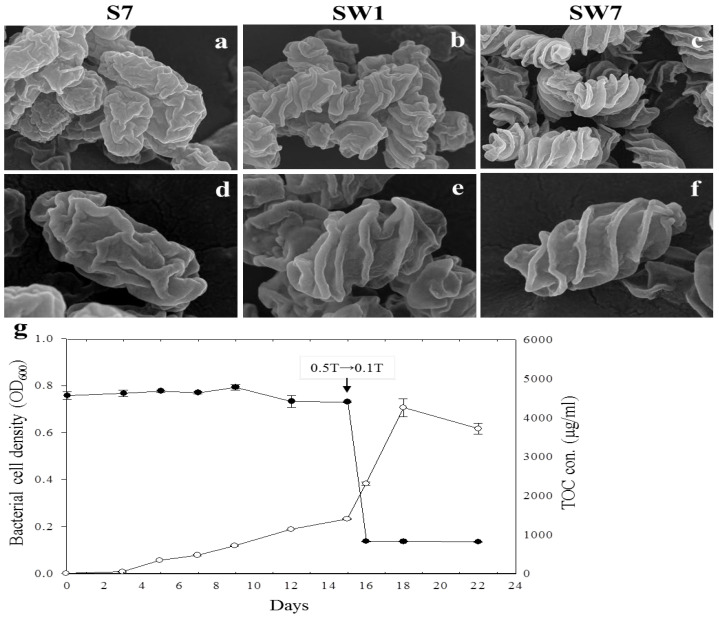
The effects of nutrient media on the growth of *Rugositalea oryzae* YC6860^T^. Bacterial cell shape was observed at 7 days after inoculation in 0.5 TSB (S7: (**a**,**d**)). The same cells were transferred to 0.1 TSB on day 15 after inoculation in 0.5 TSB and observed after 1 day (SW1: (**b**,**e**)) and 7 days (SW7: (**c**,**f**)) after transfer. The population density and the total organic carbon (TOC) were determined in cells growing in TSB media up to 22 days (**g**). The open circles (○) show cell growth while the closed circles (●) indicate TOC with time (days).

To confirm the change in cell shape from smooth to wrinkled under nutrient stress, the strain YC6860^T^ was initially cultivated in 0.5 TSB for 15 days and the 15-day-old cells were transferred to 0.1 TSB after washing with 0.1 TSB twice by centrifugation. Before the transfer, the cells collected at 7 days after inoculation (S7) in 0.5 TSB showed a smooth surface (Figure 2a,d). One day after transfer of 15-day-old cells from 0.5 TSB to 0.1 TSB, the smooth cells started to convert to wrinkled form (SW1) (Figure 2b,e). More interestingly, the grooves were more prominent at 7 days after the transfer (SW7) (Figure 2c,f). In general, more than 90% of cells exhibited wrinkling by 7 days post-transfer, while fewer than 10% retained a smooth morphology. In addition to electron microscopy, the population density of strain YC6860^T^ and TOC in culture media were measured at several time points before and after the transfer of the cultures at 15 days. The bacterial population increased slowly until 15 days in 0.5 TSB, but increased rapidly for 3 days after transfer to 0.1 TSB. However, it started decreasing at 3 days after the transfer (Figure 2g). As soil microbial communities play a crucial role in the cycling of TOC within soil ecosystems, TOC in culture media was also monitored at different time intervals to ensure transitions of nutritional levels. Indeed, the TOC in 0.5 TSB was not significantly changed until 15 days before transfer to 0.1 TSB at a range of 4200~4700 μg/mL. As expected, it was decreased to levels less than 800 μg/mL after transfer to 0.1 TSB and kept the same level until 7 days after the transfer (Figure 2g). Not only does this result provide evidence of a decrease in the nutrient concentration, but it also indicates that the nutritional transition from high to low altered bacterial shape from smooth to wrinkled form. One possibility to explain this unique change in bacterial shape could be linked to increased S/V ratios as an adaptive response to nutrient-depleted conditions for the survival of YC6860^T^ (Figure 2g). A higher S/V ratio can accommodate more high-affinity transport proteins in the cell membrane, enhancing the uptake of diffusion-limited nutrients by efficiently capturing substrate molecules from the environment. However, we cannot exclude other possibilities, since TOC may reflect general TOC, but not necessarily bioavailable carbon.

To further characterize the surface architecture of smooth versus wrinkled cells, groove depth and spacing were measured using electron microscopy images (e.g., images shown in Supplementary Figure S1). Image analysis performed with ImageJ software (v1.53) [56] revealed that smooth cells had an average groove spacing of 64.7 ± 6.4 nm, while wrinkled cells displayed a significantly wider spacing of 122.5 ± 16.9 nm. Similarly, the average groove depth for smooth cells was 14.2 ± 2.0 nm, compared to a much greater depth of 48.8 ± 3.7 nm in wrinkled cells. These measurements were derived from five independent samples, with values reported as the standard error of the mean. Together, these findings demonstrate that wrinkled cells exhibit markedly more pronounced surface undulations than their smooth counterparts.

### 3.3. Morphological Transition Correlated with Surface Area-to-Volume (S/V) Ratio

**Figure 3:**
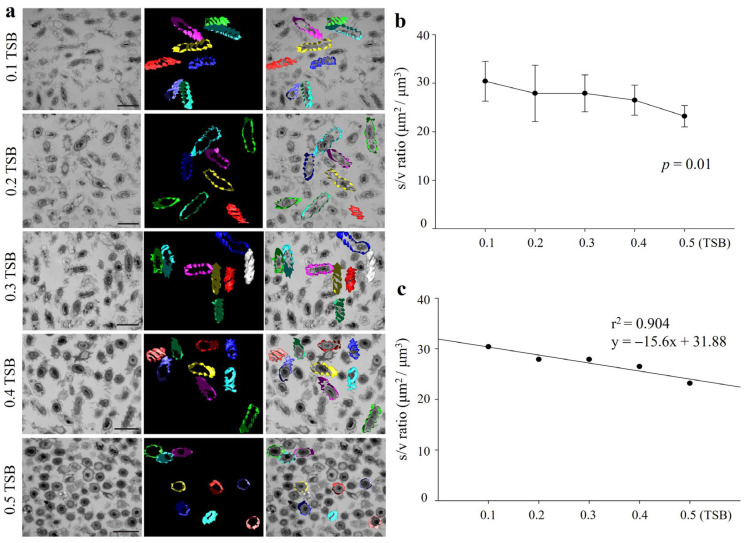
The surface-to-volume (S/V) ratio was determined in cells of *Rugositalea oryzae* YC6860^T^ grown in 0.1 to 0.5 TSB media for 15 days at 28 °C using IMOD software (**a**). The decreased S/V ratio with increasing TSB concentrations (**b**) and regression analysis (**c**). The second column of (**a**) shows three-dimensional models of cell surfaces; the models overlapped with bacterial cell images from the five TEM samples in the third column. Scale bars: 1 μm.

The S/V ratio of a bacterial cell is related to its efficiency of nutrient uptake and respiration [7,27]. To confirm the possibility of changes in S/V ratios by transition from smooth to wrinkled form, the S/V ratio of YC6860^T^ cells was analyzed at five different nutrient concentrations ranging from 0.1 to 0.5 TSB. The randomly selected cells showed altered shapes from wrinkled rods to less wrinkled and smaller cocci cells with the increase in nutrients (Figure 3a). As shown in Figure 3b,c, the higher the amount of nutrients, the lower the S/V ratios. The changes were statistically significant (*p* < 0.01) under TEM. As bacterial cells transit from smooth to wrinkled shape, they increase the S/V ratio, presumably to facilitate nutrient uptake. Regression analysis revealed strong inverse correlation between the nutrient concentration (X) and S/V ratio (Y) with the coefficient of determination r^2^ = 0.90, suggesting that with the decrease in each nutrient unit (e.g., 0.1X) in the range between 0.1 and 0.5 TSB, the S/V ratio of bacterial cells increased correspondingly during the transition (Figure 3c).

### 3.4. Phylogenetic Analysis

**Figure 4:**
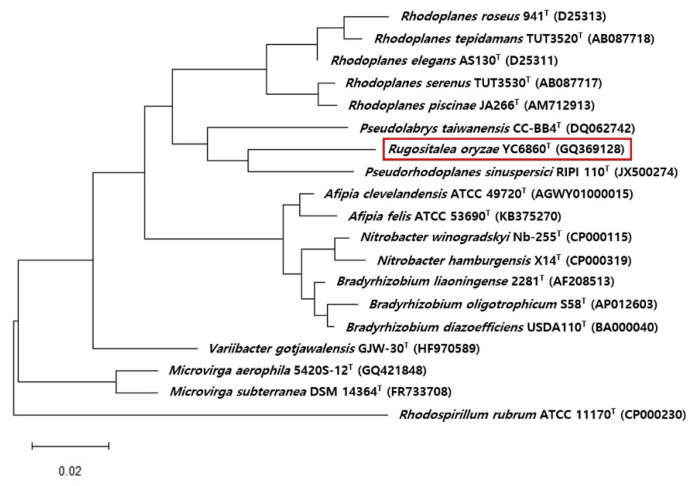
Phylogenetic tree reconstructed according to the comparative analysis of 16S rRNA gene sequences showing the relationships of *Rugositalea oryzae* YC6860^T^ (red box) and members of related genera. This phylogenetic tree was reconstructed using the maximum-likelihood method. Bar, 0.02 substitutions per nucleotide position.

The analysis of 16S rRNA gene sequence of strain YC6860^T^ (1447 bp) compared with other sequences obtained from EzBioCloud server indicated that the strain belonged to order *Rhizobiales* of class *Alphaproteobacteria.* The phylogenetic tree constructed using a neighbor-joining method and maximum-parsimony using MEGA4 software showed that the strain YC6860^T^ was phylogenetically distinct from other related taxa (Figure 4). The strain YC6860^T^ showed deep lineage with *Pseudorhodoplanes sinuspersici* RIPI 110^T^ and increased distance from *Rhodoplanes elegans* AS130^T^ with a high bootstrap value. In addition, BLAST analysis revealed that the strain YC6860^T^ was most closely related to *P. sinuspersici* RIPI 110^T^ [57], *Rhodoplanes tepidamans* TUT3520^T^ [58]^,^ and *R. elegans* AS130^T^ [59] with a similarity of 94.2%, 93.7%, and 93.7%, respectively. There was less than 93.0% similarity to other genera. Since the similarity is less than 95% at the genus level, we hypothesized that the strain YC6860^T^ could belong to a new genus.

### 3.5. Phenotypic Characterization

As strain YC6860^T^ is phylogenetically distinct from the closely related taxa based on 16S rRNA gene sequence similarity, other phenotypic traits such as morphological, biochemical, and chemotaxonomic characteristics were compared with closely related genera. Unlike the strain YC6860^T^, *P. sinuspersici* RIPI 110^T^, *R. tepidamans* TUT3520^T^, *P. taiwanensis* CC-BB4^T^, and *R. elegans* AS130^T^ were isolated from oil-contaminated soil, hot springs, soil and sludge, respectively [57,58,59,60]. Strains YC6860^T^ and *R. elegans* AS130^T^ are motile, but the others are non-motile. Salt is not necessary for their growth. The strains YC6860^T^ and *P. taiwanensis* CC-BB4^T^ are strictly aerobic, but the others are facultative anaerobes. The strain YC6860^T^ did not show any acid production or fermentation following reaction with the reagent in the API 20E kit. In API ZYM kit tests, it showed esterase (C-4), leucine arylamidase, valine arylamidase, acid phosphatase, α-fucosidase, β-glucuronidase, and α-mannosidase activities, and weak α-chymotrypsin, α-galactosidase, β-galactosidase, and N-acetyl-β-glucosaminidase activity. YC6860^T^ showed no alkaline phosphatase, lipase (C-14), or α-glucosidase activity, which differentiates it from the other strains. The DNA G + C content (63.5%) is different, while major quinone (Q-10) remains the same as in other related strains (Table 1).

### 3.6. Fatty Acid Analysis

The fatty acid profiles of YC6860^T^ and other closely related strains were compared. The fatty acids C_16:0_ and C_18:1_ w7c were both mainly detected in strains YC6860^T^, *P. sinuspersici* RIPI 110^T^, *R. tepidamans* TUT3520^T^, and *R. elegans* AS130^T^, but not in *P. taiwanensis* CC-BB4^T^ (Supplementary Table S1). Interestingly, the main fatty acid C_15:0_ anteiso in CC-BB4^T^ was absent in the other strains, including YC6860^T^. The fatty acid profiles of strain YC6860^T^ grown in 0.1 TSB and 0.5 TSB were also compared to examine the effects of different nutrient concentrations on the composition of major fatty acids. The cyclopropane fatty acid C_19:0_ cycle w8c was ~3 times higher in cells cultivated in 0.1 TSB when compared with 0.5 TSB (Table 2). This increase in cyclopropane fatty acid C_19:0_ cyclo ω8c likely reflects a stress adaptation strategy in which cyclopropane fatty acids help stabilize bacterial membranes, enhancing resistance to environmental stresses. This increase is also commonly associated with stationary-phase or slow growth states, which are often induced by nutrient scarcity. The saturated fatty acid C_10:0_ was detected only in cells in 0.5 TSB. However, the percentage of the main saturated and unsaturated fatty acids, C_16:0_ and C_18:1_ w7c, was not significantly different between the two conditions.

### 3.7. Genome Description

The low 16S rRNA gene sequence similarity, phylogenetic analysis, and other phenotypic characteristics clearly distinguish the strain YC6860^T^ from all the species in the closely related genera *Pseudorhodoplanes, Rhodoplanes* and *Pseudolabrys*. Thus, we conclude that the strain YC6860^T^ is a novel genus with a novel species in the order of *Rhizobiales* with a proposed name *Rugositalea oryzae* YC6860^T^.

The genome of *R. oryzae* YC6860^T^ comprises a single chromosome and is 8,193,889 bp long with a G + C content of 63.5% (5,205,286 bp) (Supplementary Figure S2). It is composed of a single scaffold and 12 island regions (Supplementary Figure S3). In total, 7,203,497 bp (87.91%) represents the coding capacity, and 7776 genes were identified in the genome, including 7708 (99.13%) protein-coding genes. Among the protein-coding genes identified, the functional annotation of 6069 (78.05%) genes was accomplished using different functional databases. Furthermore, 5461 (70.23%) genes matched at least a single sequence in Clusters of Orthologous Groups database (COGs). The genes are distributed into COGs functional categories (Supplementary Figure S4). Protein-coding genes connected with KEGG Orthology (KO) were 3317 (42.66%), and those connected with KEGG pathways and MetaCyc pathways were 2048 (26.34%) and 1585 (20.38%), respectively. The genome encodes 68 predicted RNAs, including 3 rRNAs, 48 tRNAs, and 17 other RNAs. No CRISPRs were found in the genome of YC6860^T^ using the CRISPRfinder. While 1290 (16.59%) coding genes were identified with signal peptides, 1745 (22.44%) genes coded transmembrane proteins. We identified 23 genes in YC6860 associated with cell shape and division. Functional analysis revealed that 17 of these genes are directly involved in bacterial cell cycle control, cell division, peptidoglycan biosynthesis, post-translational modification, and chromosome partitioning (including FtsA, FtsB, FtsZ, FtsQ, FtsX, MurJ, YceG). Additionally, three genes are implicated in cell wall remodeling, which indirectly influences cell shape, growth, and division. The remaining three genes have putative roles in cell shape and division based on conserved domain analysis and functional predictions. The presence and expression of these genes may contribute to the morphological changes observed under nutrient-limited conditions, reflecting an adaptive response of the strain to environmental stress. Nucleotide and gene counts of the *R. oryzae* YC6860^T^ genome are summarized in Supplementary Table S2.

## 4. Discussion

The main finding of this study is that *Rugositalea oryzae* YC6860^T^ alters its surface morphology from a smooth to a wrinkled form in order to increase the S/V ratio under nutrient-limited conditions, thereby enhancing its ecological fitness. This type of morphological change in bacteria, known as adaptive morphogenesis, plays an important role in survival and nutrient uptake, particularly under environmental stress. Such changes allow bacteria to optimize their shape and physiology, which might result in improving nutrient acquisition and resistance to environmental stressors, including temperature fluctuations, antibiotic exposure, and nutrient scarcity. The shape and size vary with environmental conditions of bacterial transition from high to low nutrient levels [7,15]. The novel strain YC6860^T^ was isolated from the rhizosphere of rice, with fluctuating amounts of root exudates, which affect bacterial community and growth under different tillage systems [33]. The strain was rod-shaped with small irregular wrinkles initially in general media such as 0.5 TSA or 0.5 LB. However, when the strain was grown in more diluted low-nutrient media, its cell surface morphology changed from small irregular wrinkles to large regular wrinkles. Due to the complex natural conditions of the rhizosphere in terms of nutrient composition and microbial interactions, we used sterilized defined media that simulated different nutrient concentrations and negated microbial competition in the rhizosphere to investigate the effect of nutrients on the morphological changes in this strain [6,61]. The strain YC6860^T^ grew slowly in all the tested media compared with other commonly isolated bacteria from the rhizosphere. In 0.5 TSB, it showed a normal growth curve until 15 days with most of the smooth surface cells displaying irregular form. However, in 0.1 TSB its growth reached a stationary phase after 7 days with wrinkled cell surface, which might be attributed to nutrient limitation.

Such morphological changes under different environmental conditions have also been reported in other bacterial species, such as *Actinomyces israelis*, *Arthrobacter globiformis*, *Clostridium welchii*, and a few *Pseudomonas* species, which are known to form the filamentous structure in nutrient-deficient media [7]. Similarly, a bacterial species, *Caulobacter crescentus*, in low-phosphate media showed stalk elongation due to the presence of a high-affinity phosphate-binding protein (Psts) for phosphate uptake and hydrolysis [62]. For confirmation of the shape change in this strain under nutrient limitation, the cells cultivated in 0.5 TSB were transferred to 0.1 TSB. The shape of the cells was gradually changed from fewer wrinkles to more conspicuous wrinkle formation with time, which was induced by decreased nutrient concentration. During the culture under different nutrient concentrations, the altered cell shape could reflect the adaptive behavior of the strain YC6860^T^ under nutritional stress as all other factors remained constant [2,7]. The bacterial cell density in the low-nutrient media decreased with time, and the TOC also reduced. Low nutrient levels have been reported to slow down cell division and reduce average cell size [16]. However, in this study, cells developed prominent and uniformly distributed surface wrinkles under limited-nutrient conditions, accompanied by elongation and increased S/V ratios. Therefore, we investigated the relationship between nutrient concentrations and S/V ratios using TEM. Results suggested a significantly inverse relationship between the rugosity of the cells and nutrient concentrations. One of strategies of the bacterial species to cope with nutrient-limiting conditions is to increase the S/V ratio for better access to available nutrients [7]. *C. crescentus* is the most extensively studied bacterium with an elongated stalk, which increases its cell surface area but with little effect on the cell’s S/V ratio [14,32]. TEM analysis showed that rugosity occurred throughout the cell structure, including inner and outer membrane of strain YC6860^T^ that increased the S/V ratio of the cells with decreasing nutrient concentration, which resulted in an increase in the space available for nutrient absorption under nutrient-limiting conditions. The association between rugosity and nutrient absorption presents a compelling hypothesis; however, direct functional validation should be conducted in future studies to substantiate it.

Based on 16S rRNA gene sequence analysis, the strain YC6860^T^ was found to belong to the order *Rhizobiales*, which is a phenotypically heterogeneous assemblage of Gram-negative bacteria [63]. As the strain showed low sequence similarity (<94.3%) to the closely related members of genera *Pseudorhodoplanes, Rhodoplanes,* and *Pseudolabrys*, it should be assigned a novel genus, with a name proposed as *Rugositalea* and a representative species *R. oryzae*. The physiological and biochemical characteristics of this strain also showed its distinct features from other related species. For validation of the new strain, its name should be added into the list of *International Journal of Systematic and Evolutionary Microbiology* (IJSEM).

Morphological adaptation may also be influenced by changes in membrane fatty acid composition, as reflected in the fatty acid profiles of strain YC6860^T^ at different nutrient media. The fatty acid profile in strain YC6860^T^ at low-nutrient media was distinct when compared with high-nutrient media, suggesting that the strain also responds to low-nutrient conditions by changing the fatty acid composition. However, the difference between fatty acid compositions at high- and low-nutrient media is not striking. The fatty acid profile showed a high percentage of saturated fatty acid (C_16:0_) and unsaturated fatty acid (C_18:1_ w7c) levels in YC6860^T^, *Pseudorhodoplanes* and *R. elegans* AS130^T^, but not in *P. taiwanensis* CC-BB4^T^. The fatty acid composition of bacterial species is modified by the environmental changes, especially pH, temperature, and the nutrients of growth media [64]. The alteration of cell size due to nutrient limitation is also caused by fatty acid biosynthesis, which controls cell expansion and permeability [28].

The full genome of this novel strain was sequenced and deposited. As the strain YC6860^T^ exhibits extraordinary shapes depending on nutrient concentrations, the genome data might provide an opportunity to study the presence of unique metabolic pathways previously unknown for the morphological evolution of bacteria [32]. To further explore the genetic uniqueness of strain YC6860^T^, a comparative genomic approach was employed using the Phylogenetic Profiler for Single Genes tool in IMG/MER against 18 strains, including YC6860^T^ and other phylogenetically related genera from Figure 4. This initial screening yielded 1000 genes predicted to be specific to YC6860 ^T^. To further validate these findings, the nucleotide sequences of these candidate genes were subjected to BLASTn analysis against the NCBI database using a command-line pipeline. Genes showing hits exclusively to YC6860^T^ with no significant alignments to other organisms were considered strain-specific. After rigorous filtering, we uncovered 390 high-confidence genes unique to this strain, such as a PHB depolymerase-like protein, an S1-C subfamily serine protease, a phasin protein, and a CBS-domain protein, which stand out as promising molecular markers and unique candidates for future investigations.

This study demonstrated only the influence of nutrients on the cell shape of the pleomorphic strain YC6860^T^ comprising smooth and wrinkled cells. Under limited nutrient conditions, the growth rate of this strain declined due to reduced cell division; however, increased groove depth and spacing on the cell surface contributed to a higher S/V ratio, indicating that bacteria manipulated the morphology to their advantage in a given environment [7,16]. Surprisingly, star-shaped cells similar to the wrinkled cell of strain YC6860^T^ were previously observed in the concentrated cell fluid of natural soil by TEM [65]. It was discussed that the possibility of an authentic form for certain soil microorganisms could not be ruled out. It is not known how rugosity of the cell is formed in response to nutrient limitation. Although the precise mechanisms underlying bacterial shape evolution remain unclear, it is speculated that actin-like cytoplasmic proteins (e.g., MerB, Mb1, and MreBH), scaffold proteins (e.g., FtsZ, FtsA, and ZipA), and peptidoglycan synthesis proteins (e.g., Mur family) play key roles in determining cell shape and size [30,66,67]. Thus, the mechanism involved in cell shape in this strain needs to be elucidated with further investigations into the molecular ecology. Additionally, in relation to envelope remodeling and cell wall modification, the PHB depolymerase-like protein may mobilize carbon reserves under nutrient stress, while CBS-domain proteins, as energy sensors, may regulate enzymes involved in shape modulation. Together, these genes suggest mechanistic roles in the morphological plasticity of strain YC6860^T^. Some of the unique genes identified in strain YC6860^T^ are also associated with morphogenesis and envelope biosynthesis. Included are genes involved in peptidoglycan synthesis (*murJ*), cell division (*ftsZ*, *ftsQ*, *ftsX*), and cytoskeletal regulation (*mreB*), supporting a genomic basis for the observed morphological changes under nutrient stress.

The ecological significance of *Rugositalea oryzae* in the rice rhizosphere is not yet fully understood, particularly regarding the functional implications of its morphological plasticity. The observed transition from smooth to wrinkled rod-shaped cells under nutrient-limited conditions may represent an adaptive strategy that enhances survival and competitiveness in the complex rhizosphere environment. This morphological change could influence microbial interactions by affecting surface adhesion, biofilm formation, or spatial organization within the rhizosphere microbiome. Additionally, the altered cell surface structure may impact host recognition or plant–microbe signaling processes, potentially modulating colonization dynamics or eliciting specific responses from the host plant. These interactions could have downstream effects on nutrient cycling, plant health, or microbial community composition in the rhizosphere. Therefore, future studies should aim to characterize the functional outcomes of this plasticity through in situ experiments, co-culture assays, transcriptomics, CRISPR mutagenesis, and proteomic or metabolomic profiling under greenhouse or field conditions. Such investigations will provide valuable insight into whether morphological adaptability confers ecological advantages to *R. oryzae* and contributes to its role in shaping the microbial ecology of the rice rhizosphere.

*Rugositalea* (Ru.go’si.tal e.a. L. adj. *rugosus* of rugose or wrinkled; L. fem. n. *talea* a rod; N.L. fem. n. *Rugositalea* a rugose forming rod) cells are aerobic, Gram-negative and motile with a polar flagellum (Supplementary Figure S5). The morphology is pleomorphic ranging from rod to cocci under different nutrient concentrations. Under limited-nutrient conditions, the cells changed from rod to wrinkled shape with rugosity on the cell surface, reflecting restructured cell wall and membrane. The cells tested are positive for oxidase and catalase. The respiratory quinone is ubiquinone Q-10. The major fatty acids are C_18:1_ w7c and C_16:0_. *Rugositalea* belongs to the *Bradyrhizobiaceae* family within the order *Rhizobiales*. The type species is *Rugositalea oryzae*.

The main features of *Rugositalea oryzae* YC6860^T^ (o.ry za.e. L. fem. n. *Oryza* genus name of rice; L. gen. n. *oryzae* of rice, refers to the isolation of type strain from a rice field) are similar to those described for the genus: creamy, smooth, flat and circular colonies, 0.1–0.5 mm in diameter and with entire margins, formed on 0.1 TSA after 30 days at 28 °C. Cells measure 0.4–0.6 × 1.1–1.9 μm, divided by binary fission in the mid-exponential phase. The temperature range for growth is 16–30 °C (optimum 28 °C). The growth pH range is 6–10 (optimum 7). It does not require NaCl for growth, but cannot tolerate NaCl above 0.5% (*w*/*v*). It exhibits esterase (C-4), leucine arylamidase, acid phosphatase, napthol-AS-BI-phosphohydrolase, α-fucosidase, valine arylamidase, β-glucuronidase, and α-mannosidase activities, and weak activity of β-galactosidase, β-glucosidase, α-chymotrypsin, α-galatosidase and N-acetyl-β-glucosaminidase. Alkaline phosphatase, lipase (C14), and α-glucosidase are negative. It grows on minimal agar medium supplemented with a small amount (0.3%, *w*/*v*) of inositol, melibiose, lactose, salicine, raffinose, glucose, sucrose, and mannose as a single carbon source. However, it cannot grow in media containing fructose, mannitol, and arabinose. It hydrolyzed hippurate but not chitin, casein, or xylan. Nitrate is reduced to nitrite or nitrogen. The G + C content of the type strain is 63.5 mol%. The type strain, YC6860^T^ (=KCCM 43037^T^ = NBRC^T^ 109316), was isolated from the rhizosphere of no-tilled rice field at Jinju, Korea.

## 5. Conclusions

The wrinkled bacterium newly discovered in the rice rhizosphere has a unique shape that has not been reported in bacterial morphology to date. The novelty and importance findings of this study include the identification of wrinkle formation as a potential adaptation to nutrient-limited environments, and the associated increase in the S/V ratio that may enable successful bacterial survival in the rhizosphere. To the best of our knowledge, this is the first report of wrinkle formation in bacteria; however, the underlying molecular mechanisms have yet to be elucidated. Further molecular studies are needed to elucidate the basis of this morphological trait, particularly in relation to biofilm formation, quorum sensing, the complex environmental conditions of plant root systems, and plant–microbe interactions in the rhizosphere. If novel functions are revealed, this bacterium could have potential applications in addressing challenges such as climate change and antibiotic resistance.

## Figures and Tables

**Table 1:** Phenotypic variation in *Rugositalea oryzae* YC6860^T^ and phylogenetically related genera in the order *Rhizobiales*. Taxa: *Rugositalea oryzae* YC6860^T^; *Pseudorhodoplanes sinuspersici* RIPI 110^T^ [57]; *Rhodoplanes tepidamans* TUT3520^T^ [58]; *Rhodoplanes elegans* AS130^T^ [59]; *Pseudolabrys taiwanensis* CC-BB4^T^ [60]. ND, not determined; +, positive; -, negative.

Characteristics	*Rugositalea oryzae* YC6860^T^	*Pseudorhodoplanes sinuspersici* RIPI 110^T^	*Rhodoplanes tepidamans* TUT3520^T^	*Rhodoplanes elegans* AS130^T^	*Pseudolabrys taiwanensis* CC-BB4^T^
Isolation source	Rice rhizosphere	Oil-contaminated Soil site	Hot springs	Sludge	Soil
Gram-staining	Negative	Negative	Negative	Negative	Negative
Motility	Motile	Non-motile	Motile	Motile	Non-motile
NaCl requirement	0–1%	0–3.5%	0%	<1%	ND
Oxidase/Catalase	+/+	+/+	ND	ND	+/+
Temperature (°C)	16–30	15–35	40	30–35 (optimal)	15–36
pH	6.0–10.0	5.5–8.0	6.8–7.5	6–8.5	4.2–8.5
Aerobic/Anaerobic condition	+/-	+/+	+/+	+/+	+/-
Alkaline phosphatase	-	ND	ND	+	+
Lipase (C14)	-	ND	ND	-	+
DNA G + C content (%)	63.5	59.4	69.9	69.7	67.0
Major quinone	Q-10	Q-10	Q-10, R-10	Q-10, RQ-10	ND

Data for the related type strains are from this study except *P. sinuspersici* RIPI 110^T^ [57] and *R. tepidamans* TUT3520^T^ [58].

**Table 2:** The cellular fatty acid profile of *Rugositalea oryzae* YC6860^T^ grown in TSB media at different nutrient concentrations. Data were expressed as percentages of fatty acid determined after cultivating the strain YC6860^T^ at 28 °C for 10 days.

Fatty Acids (%)	0.1 TSB	0.5 TSB
Saturated		
C_10:0_	-	4.02
C_12:0_	1.37	4.62
C_14:0_	0.78	0.98
C_16:0_	18.93	19.78
C_17:0_	0.93	0.77
C_18:0_	1.38	1.41
Unsaturated		
C_17:1_w8c	0.73	-
C_18:1_w5c	-	0.1
C_18:1_w7c Hydroxy	55.18	58.33
C_16:1_ 2OH	0.17	-
C_18:0_ 3OH	0.56	0.41
Cyclopropane		
C_17:0_ CYCLO	1.33	0.51
C_19:0_ CYCLO w8c	12.2	4.8

## Data Availability

The original contributions presented in this study are included in the article/Supplementary Material. Further inquiries can be directed to the corresponding authors.

## Supplementary Materials

The following are available online at https://www.mdpi.com/article/10.3390/life15091337/s1. Table S1. Cellular fatty acid profiles of *Rugositalea oryzae* YC6860^T^ and phylogenetically related genera in the order *Rhizobiales*. Taxa: *Rugositalea oryzae* YC6860^T^; *Pseudorhodoplanes sinuspersici* RIPI 110^T^; *Rhodoplanes tepidamans* TUT3520^T^; *Rhodoplanes elegans* AS130^T^; *Pseudolabrys taiwanensis* CC-BB4^T^. Table S2. Nucleotide content and gene count levels of the *Rugositalea oryzae* YC6860^T^ genome. Figure S1. The growth of *Rugositalea oryzae* YC6860^T^ cells grown on (**a**) 0.1 LB and (**b**) 0.5 LB at 28 °C in a rotary shaker (50 rpm) for 5 days. Figure S2. Circular representation of the *Rugositalea oryzae* YC6860^T^ genome. Figure S3. Map of the island like regions in the *Rugositalea oryzae* YC6860^T^ genome. Figure S4. The distribution of genes into COGs functional categories of the *Rugositalea oryzae* YC6860^T^ genome. Figure S5. Cells of *Rugositalea oryzae* YC6860^T^ with a polar flagellum grown in 0.1 TSB at 28 °C in a rotary shaker (50 rpm) for 5 days.
