# Supplementary material for: Unusual Morphological Changes of a Novel Wrinkled Bacterium Isolated from the Rice Rhizosphere Under Nutrient Stress
Source: Life (Basel). 2025 Aug 23;15(9):1337. doi: 10.3390/life15091337 (PMC12470966; doi:10.3390/life15091337)
Supplement: Supplementary file 1 [file life-15-01337-s001.zip › life-3762949-supplementary.pdf]

SUPPLEMENTAL MATERIALS

Table S1. Cellular fatty acid profiles of *Rugositalea oryzae* YC6860^T and phylogenetically related genera in the order *Rhizobiales.* Taxa: *Rugositalea oryzae* YC6860^T; *Pseudorhodoplanes sinuspersici* RIPI 110^T; *Rhodoplanes tepidamans* TUT3520^T; *Rhodoplanes elegans* AS130^T; *Pseudolabrys taiwanensis* CC-BB4^T.

Fatty acids (%)	*Rugositalea oryzae* YC6860^T	*Pseudorhodoplanes sinuspersici* RIPI 110^T	*Rhodoplanes tepidamans* TUT3520^T	*Rhodoplanes elegans* AS130^T	*Pseudolabrys taiwanensis* CC-BB4^T
Saturated					
$C_{12:0}$	1.05	-	-	-	1.25
$C_{14:0}$	2.48	-	-	3.00	2.56
$C_{14:0}$ iso	-	-	-	-	4.87
$C_{15:0}$ iso	-	-	-	-	6.42
$C_{15:0}$ anteiso	-	-	-	-	33.35
$C_{16:0}$	30.29	11.00	10.7-17.5	25.91	-
$C_{16:0}$ iso	-	-	-	-	3.33
$C_{17:0}$	-	-	-	-	0.69
$C_{17:0}$ iso	-	2.80	-	-	1.74
$C_{17:0}$ anteiso	-	-	-	-	4.26
$C_{18:0}$	-	2.30	-	3.09	14.54
$C_{18:0}$ iso	-	-	-	-	1.25
$C_{19:0}$	-	-	-	-	0.59
$C_{19:0}$ iso	-	-	-	-	0.47
$C_{20:0}$	-	-	-	-	13.31
Unsaturated					
$C_{14:1}$w5c	1.27	-	-	0.99	0.49
$C_{16:1}$w5c	-	-	-	4.55	-
$C_{16:1}$w7c/$C_{16:1}$ w6c	1.83	-	-	2.63	-
$C_{18:1}$w7c	57.11	-	74-80	58.83	-
Cyclopropane					
$C_{19:0}$ cyclo w8c	4.59	10.00	-	-	-

Methylated					
$C_{16:0}$ 10-methyl	-	-	-	-	0.72
Summed feature 8		72.90			

summed feature 8 comprises $C_{18:1}$ w7c and/or $C_{18:1}$ w6c.

Data for the type strains of *Rugositalea oryzae* YC6860^T, *Rhodoplanes elegans* AS130^T and *Pseudolabrys taiwanensis* CC-BB4^T were determined after cultivating the strains in R2A at 28°C for 10 days from this study.

Data for the other related type strains *P. sinuspersici* RIPI 110^T and *R. tepidamans* TUT3520^T were determined after growing the strains in R2A and RPM media at 30°C for 5 days, respectively.

Table S2. Nucleotide content and gene count levels of the *Rugositalea oryzae* YC6860^T genome.

Attribute	Value	% of Total
DNA, total number of bases	8193889	100.00
DNA coding number of bases	7203497	87.91
DNA G+C number of bases	5205286	63.53
DNA scaffolds	1	100.00
Genes total number	7776	100.00
Protein coding genes	7708	99.13
RNA genes	68	0.87
Protein coding genes with function prediction	6069	78.05
Protein coding genes with enzymes	1766	22.71
Protein coding genes connected to KEGG pathways	2048	26.34
Protein coding genes connected to KEGG Orthology (KO)	3317	42.66
Protein coding genes connected to MetaCyc pathways	1585	20.38
Protein coding genes with COGs	5461	70.23
Protein coding genes coding signal peptides	1290	16.59
Protein coding genes coding transmembrane proteins	1745	22.44
CRISPR repeats	0	0.00

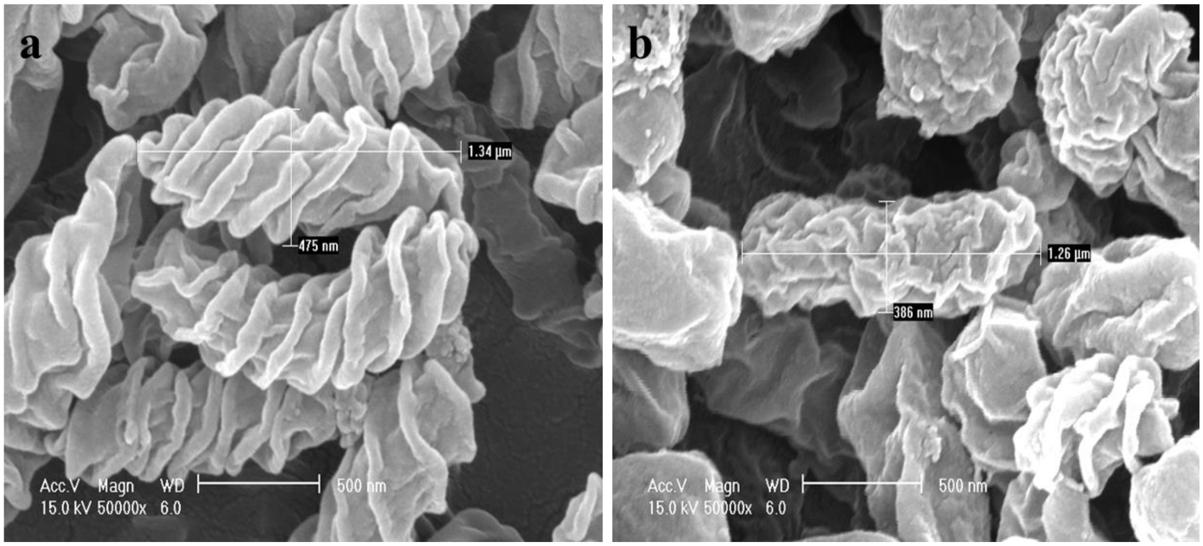

Figure S1. The growth of *Rugositalea oryzae* YC6860^{T} cells grown on (a) 0.1 LB and (b) 0.5 LB broth at 28°C in a rotary shaker (50 rpm) for 5 days.

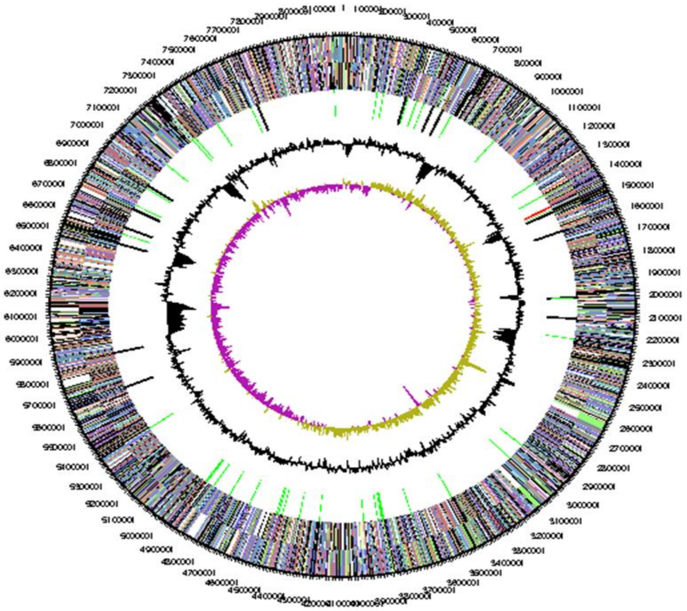

Figure S2. Circular representation of the *Rugositalea oryzae* YC6860^{T} genome. Circles from the outside to the center: genes on forward strand colored by COGs categories, genes on reverse strand

(colored by COG categories), tRNA (green), rRNA (red), other RNAs (black), GC content and GC skew.

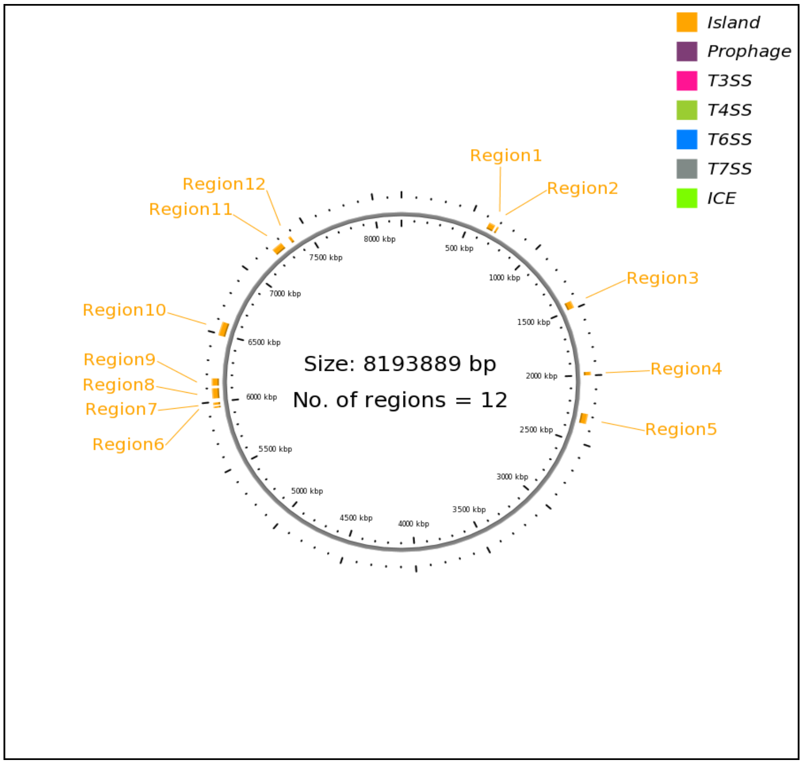

Figure S3. Map of the island like regions in the *Rugositalea oryzae* YC6860^{T} genome.

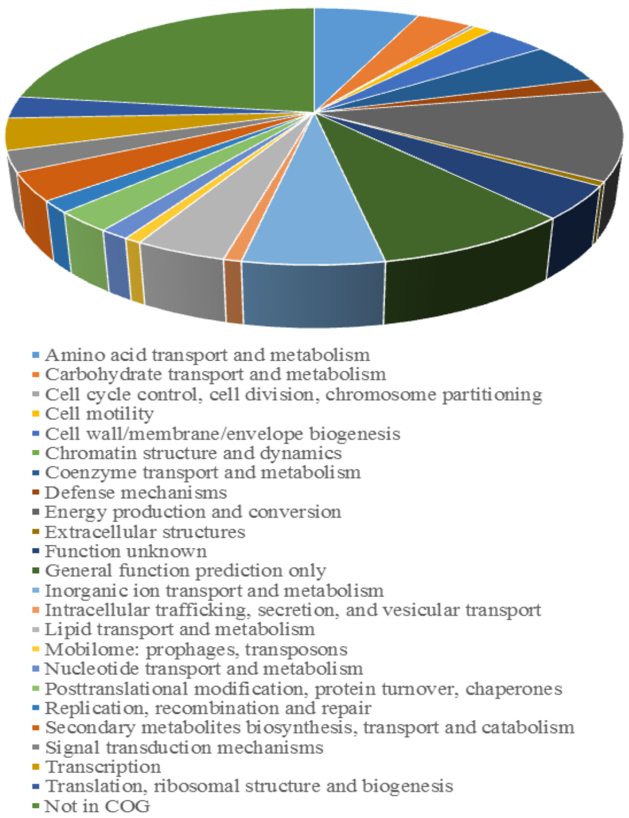

Figure S4. The distribution of genes into COGs functional categories of the *Rugositalea oryzae* YC6860^{T} genome.

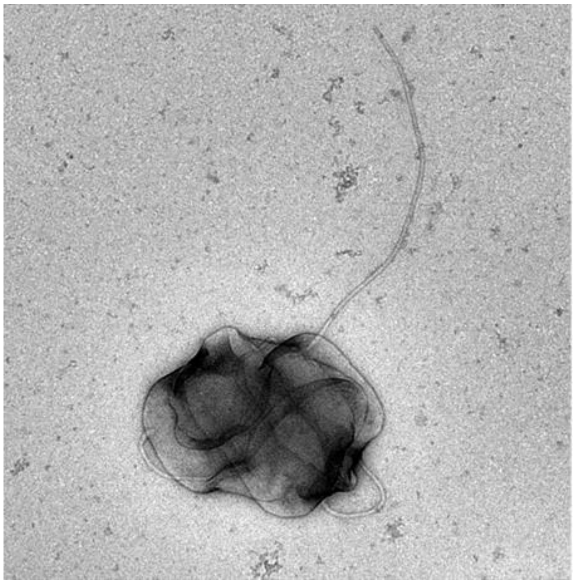

Figure S5. Cells of *Rugositalea oryzae* YC6860^{T} with a polar flagellum grown in 0.1 TSB at 28°C in a rotary shaker (50 rpm) for 5 days.
